# Undifferentiated laryngeal carcinoma with hyaline bodies in a cat

**DOI:** 10.1186/s13028-021-00613-y

**Published:** 2021-11-22

**Authors:** Filippo Torrigiani, Maria Elena Gelain, Laura Cavicchioli, Roberta Di Maggio, Tommaso Banzato, Federico Bonsembiante

**Affiliations:** 1 Department of Comparative Biomedicine and Food Science, University of Padua, 35020 Legnaro, Padua Italy; 2 Department of Animal Medicine, Productions and Health, University of Padua, 35020 Legnaro, Padua Italy

**Keywords:** Carcinoma, Cytology, Immunohistochemistry, Larynx, Pancytokeratin, Transmission electron microscopy

## Abstract

**Background:**

Primary laryngeal neoplasms are rare in cats, with lymphoma and squamous cell carcinoma being the most commonly diagnosed tumour types. These tumours are usually highly aggressive, difficult to treat, and have a poor prognosis. Here an undifferentiated laryngeal carcinoma with hyaline bodies in a cat is reported.

**Case presentation:**

A 13-year-old cat was presented for progressive respiratory signs. Diagnostic procedures revealed a partially obstructive laryngeal mass. Cytology was compatible with a poorly differentiated malignant tumour, with neoplastic cells frequently containing large intracytoplasmic hyaline bodies. After 1 month the patient was euthanised due to a worsening clinical condition and submitted for post-mortem examination, which confirmed the presence of two laryngeal masses. Histopathology confirmed the presence of an undifferentiated neoplasm with marked features of malignancy. Strong immunolabelling for pancytokeratin led to a diagnosis of undifferentiated carcinoma, however, histochemical and immunohistochemical investigations could not elucidate the origin of the large intracytoplasmic hyaline bodies observed in tumour cells, which appeared as non-membrane bound deposits of electron-dense material on transmission electron microscopy.

**Conclusion:**

This is the first report of primary undifferentiated laryngeal carcinoma in a cat. Our case confirms the clinical features and the short survival that have been reported in other studies describing feline laryngeal tumours. Moreover, for the first time in feline literature, we describe the presence of intracytoplasmic hyaline bodies in neoplastic cells that were compatible with the so-called hyaline granules reported in different human cancers and also in the dog.

## Background

Laryngeal tumours are rare in companion animals. In a 10-year survey conducted on biopsy and necropsy specimens, laryngeal tumours accounted for 0.2% of canine cases and 0.14% of feline cases, respectively [1]. The most commonly diagnosed laryngeal neoplasms in cats are lymphoma (which represents up to 50% of feline primary laryngeal tumours) and squamous cell carcinoma, with adenocarcinoma and undifferentiated round cell tumours being less represented [2–7]. Affected cats are usually presented with severe respiratory signs such as dyspnoea, stridor, and coughing. Regardless of the tumour type, primary laryngeal neoplasms are usually highly aggressive and poorly responsive to therapy. Treatment options for laryngeal lymphoma include chemotherapy, radiation therapy and tracheostomy, or combinations of these, while carcinomas have been treated with permanent tracheostomy or palliative treatment such as prednisolone or COX2 inhibitors [2–5, 7, 8]. In most studies, however, cats are usually euthanised at diagnosis due to the severity of symptoms, the invasiveness of some treatment procedures (i.e., tracheotomy, permanent tracheostomy), and the poor prognosis [2–4]. Reported survival times for feline laryngeal neoplasm are usually low and range from a median of 1 day in untreated patients to medians of 134.5–150 days in patients with lymphoma treated with multimodal therapy [2–4]. Here we describe the first reported case of primary undifferentiated carcinoma of the larynx in a cat and characterize intracytoplasmic hyaline bodies observed within tumour cells.

## Case presentation

A 13-year-old spayed female domestic shorthair cat was presented to the Veterinary Teaching Hospital of the University of Padua with a recent history of weight loss, progressive dyspnoea, respiratory crises and multiple episodes of regurgitation in the previous week. According to the owners, the cat also experienced difficulty in eating and drinking.

On physical examination, the patient had decreased muscular masses with a reduced body condition score (BCS 4/9), mucus membranes appeared slightly pale with capillary refill time of 2 s. The patient exhibited marked tachypnoea (65 breaths/min), with inspiratory dyspnoea and bilateral reinforced respiratory sound with stridors and wheezes on thoracic auscultation, and slight bradycardia (140 bpm).

Diagnostic workup included haematology, biochemistry and urinalysis, which were within normal limits, and serum protein electrophoresis that revealed a slight decrease in the albumin fraction and an increase in the α2- and β1-globulin fractions.

Head, neck and thoracic x-rays performed under sedation revealed a soft tissue 2.6 × 2.2 cm mass that displaced the larynx dorsally. Moreover, a mild pulmonary interstitial pattern was observed bilaterally.

**Fig. 1:**
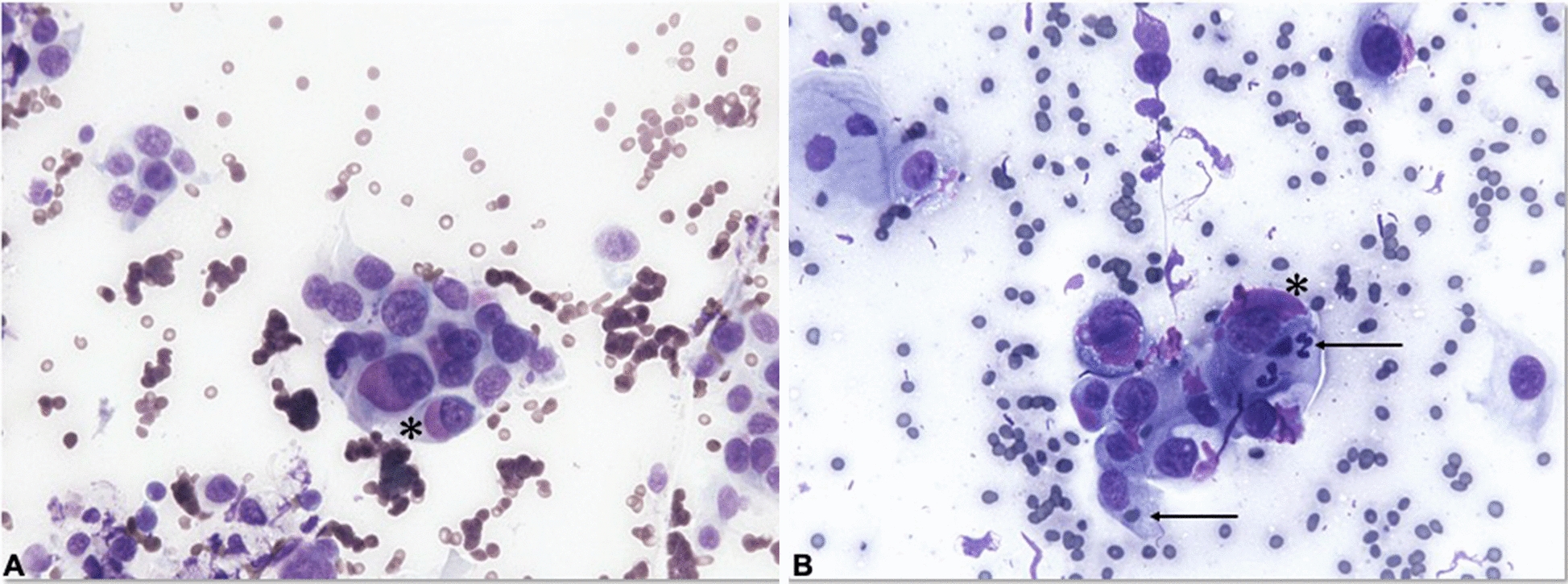
Trans-oral fine needle aspiration cytology: **A** groups of highly pleomorphic neoplastic cells with marked anisokaryosis and multinucleation, often containing one large, deeply eosinophilic, perinuclear body (asterisk). **B** Cellular cannibalism: neoplastic cells engulfing cell types of different lineages (i.e. erythrocytes and neutrophils) (arrows). The large perinuclear bodies were consistently observed in cytological samples (asterisk). May Grunwald Giemsa stain, ×400

The laryngeal mass was inspected under sedation by oral cavity examination and sampled for cytology through a trans-oral fine needle aspiration. Cytology revealed a highly pleomorphic cell population. Cells were polygonal or elongated in shape, organized in small groups or individually, with abundant, lightly basophilic cytoplasm that often showed one large, intensely eosinophilic, perinuclear body. Nuclei were round to irregular in shape with granular chromatin and prominent nucleoli. Anisocytosis and anisokaryosis were marked. Additionally, macrokaryosis and multinucleated cells as well as cellular cannibalism were frequently observed (Fig. 1). Based on the cytological findings, diagnosis of a malignant, poorly differentiated neoplasm was made. Based on cytomorphology, the differential diagnoses included a poorly differentiated carcinoma and a poorly differentiated soft tissue sarcoma.

**Fig. 2:**
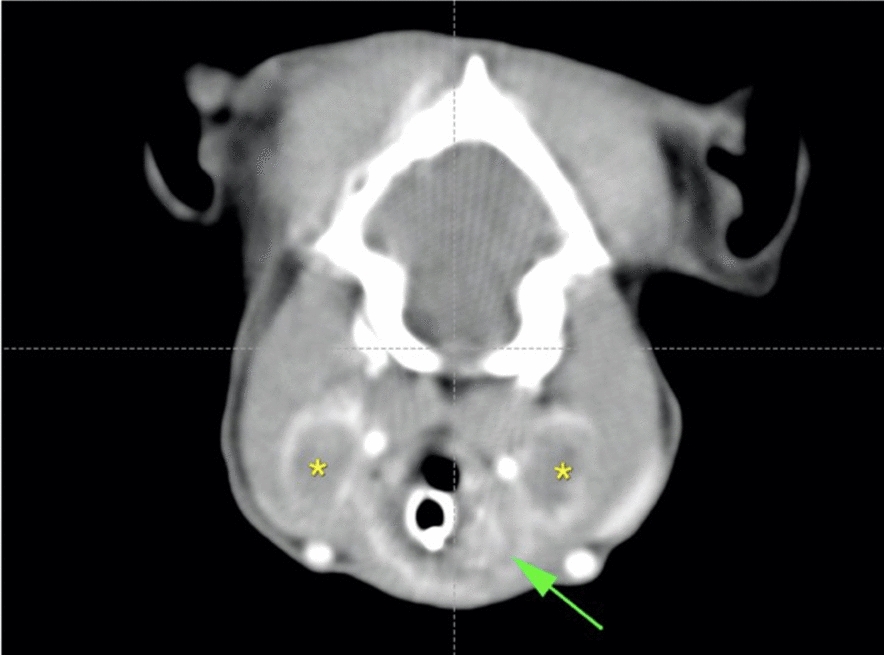
Contrast enhanced CT scan of the head: post contrast CT image of the lesion showing the moderately contrast enhancing laryngeal mass (arrow), and bilaterally enlarged retropharyngeal lymph nodes (asterisks)

Following the cytology results, a contrast-enhanced CT scan of the head, thorax and abdomen was performed for staging purposes and for surgical planning. CT findings showed an ill-defined, moderately contrast enhancing (pre-contrast HU: 36, post-contrast HU: 96) mass surrounding and largely invading the larynx. Both retropharyngeal lymph nodes appeared markedly enlarged and necrotic (Fig. 2). No further tomographic changes in distant organs were detected.

The extension of the tumour and its location were not amenable to conservative surgery with complete surgical margins. Due to poor prognosis owners declined further investigations and opted for symptomatic treatment with meloxicam 0.6 mg/kg daily.

After a partial remission of clinical signs over a period of 10 days, the patient showed progressive deterioration of clinical conditions and severe respiratory sings and was euthanized 1 month after first clinical presentation. Euthanasia was performed under general anaesthesia using a combination of a curariform-like agent, a narcotic, and a local anaesthetic.

**Fig. 3:**
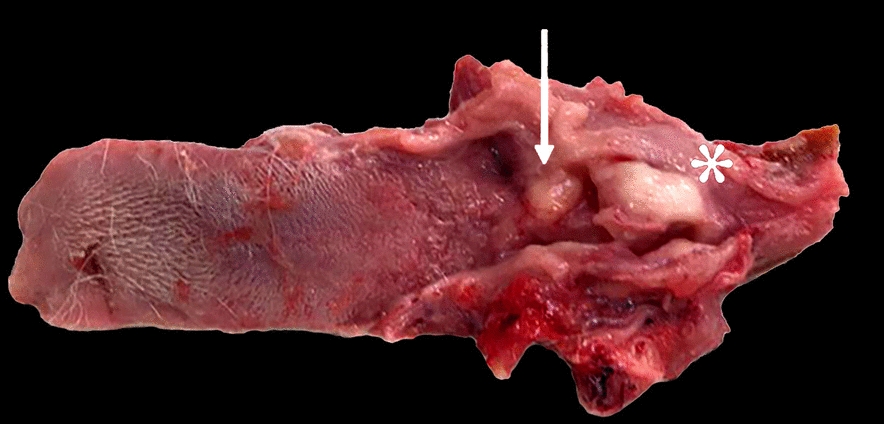
Post-mortem examination of the larynx: two distinct firm, white masses obstructing laryngeal lumen, the larger extends caudally involving the vocal cords (asterisk), the smaller mass is located more cranially and involves the aryepiglottic fold (arrow)

On post-mortem examination, two distinct, bilateral laryngeal masses were found. Both tumour masses arose from the mucosa of the cuneiform process of the arytenoid cartilage, and protruded in the laryngeal inlet dramatically reducing the lumen. The larger mass measured 2.8 × 2 cm and extended caudally involving the vocal cords, whilst the smaller was located more cranially involving the aryepiglottic fold. Both masses were well demarcated, exophytic, with firm texture, smooth, with white-tan colour on cut surface (Fig. 3). Mild hyperaemia of the surrounding structures was observed and no other alterations were found on necropsy.

Both laryngeal masses were sampled, formalin fixed, routinely processed and haematoxylin and eosin stained for histological examination.

**Fig. 4:**
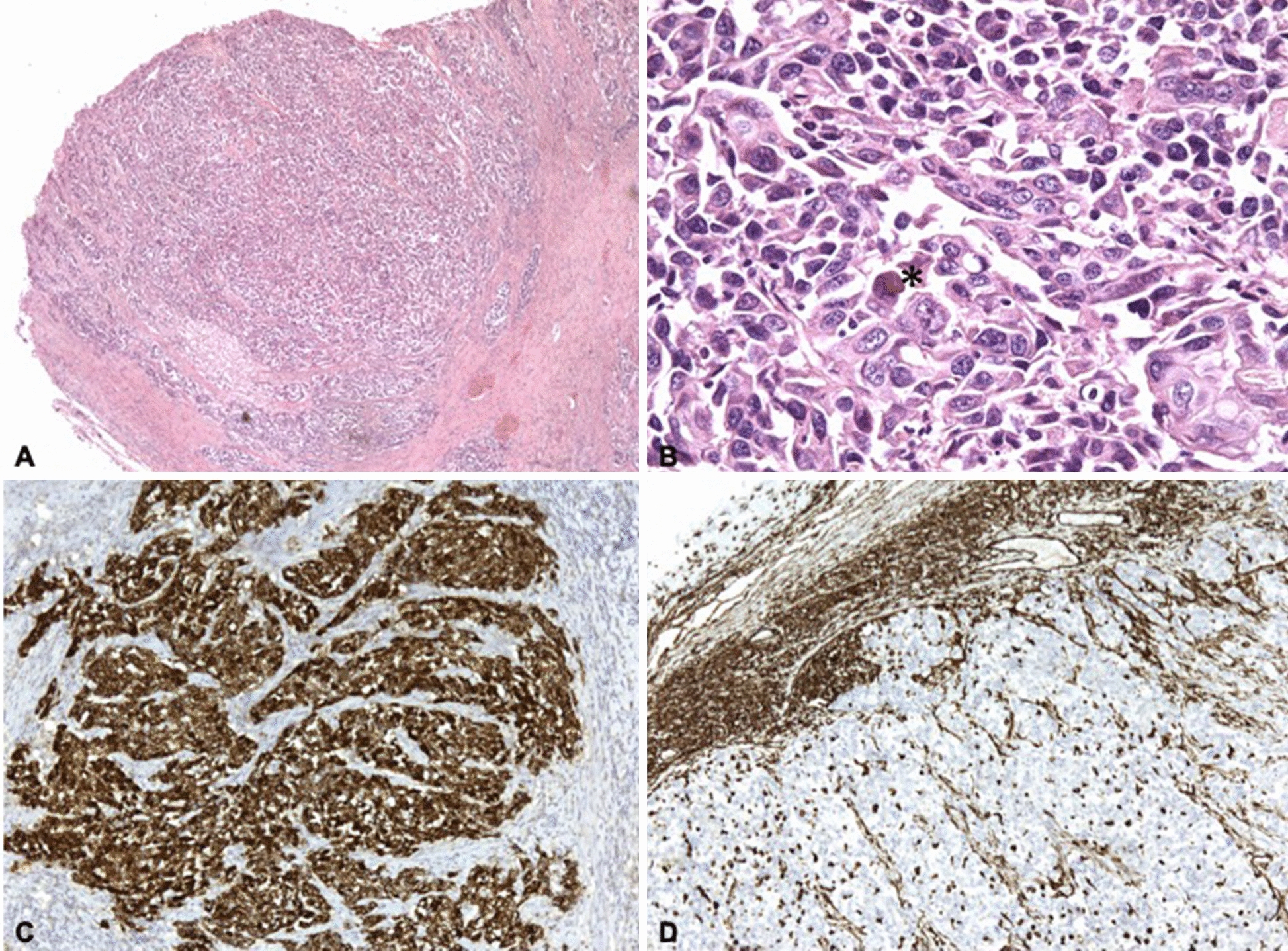
Histological and immunohistochemical features of the neoplasm: **A** neoplastic cells are arranged in densely cellular, partially encapsulated nodules and are supported by a thick fibrovascular stroma that subdivides the nodules in variably sized packets (HE stain ×40). **B** Higher magnification shows highly pleomorphic neoplastic cells multifocally presenting one large, brightly eosinophilic perinuclear body (asterisk) (HE stain ×400). **C** Immunohistochemistry for pancytokeratin, neoplastic cells diffusely show intense cytoplasmic immunolabelling for pancytokeratin (×100). **D** Immunocytochemistry for vimentin, fibrovascular stromal cells as well as tumour infiltrating inflammatory cells (lymphocytes and macrophages) diffusely show intense cytoplasmic immunolabelling for vimentin (×100)

All sections were characterised by an infiltrative neoplastic population that expanded the laryngeal submucosa and extended deeper involving the arytenoid cartilage. Neoplastic cells were observed in poorly demarcated, partially encapsulated nodules, in which they were arranged in nests and cords supported by a moderate to abundant fibrovascular stroma. Cells were moderately preserved, oval to elongated in shape, with mostly distinct borders and a variable amount of pale eosinophilic cytoplasm, with scattered basophilic granules and an occasional large (up to 20 μm), brightly eosinophilic, glassy round to oval body, mainly located in the perinuclear area. Nuclei were round to oval, occasionally indented, with coarsely stippled chromatin and one prominent nucleolus. Histological criteria of malignancy, including anisocytosis, anisokaryosis, and macrokaryosis, were marked. Multinucleated cells (up to 6 nuclei per cell), and cellular cannibalism were frequently observed, while mitotic figures were 4 per 10 high power fields (HPFs; diameter of the field of view = 0.55 mm; HPF = 237 mm^2^; 40× magnification, Nikon Eclipse Ci-L, Nikon Instruments, Japan). Lymphocytic infiltration of the tumour mass and peripheral aggregates of lymphocytes with rare histiocytes were also present (Fig. 4).

Based on histology, a diagnosis of undifferentiated malignant laryngeal tumour was made and any possible cellular origin of the tumour between epithelial, mesenchymal, or neuroendocrine was included. In order to further investigate the tumour phenotype, an immunohistochemistry (IHC) panel including pancytokeratin (PanCK), cytokeratin 5/6 (CK 5/6), and cytokeratin 8/18 (CK 8/18) as epithelial markers, p63 and calponin as myoepithelial markers, vimentin as mesenchymal marker, neurofilaments and chromogranin as neuroendocrine markers was performed, while CD3 and CD20 were included to better characterise the lymphocytic infiltrate. All antibodies included in the IHC panel were previously tested in cats (Table 1).

**Table 1:** Antibodies used in this study and related features

Target	Marker	Clone	Dilution	Secondary antibody	Manufacturer
Epithelial markers	panCK	AE1/AE3	1:100	Mouse	Dakocytomation
	CK8/18	5D3	1:30	Mouse	Novocastra
	CK5/6	D5/I6 B4	1:50	Mouse	Invitrogen
Myoepithelial markers	p63	4A4	1:200	Mouse	Santa Cruz Biotechnology, Inc
	Calponin	Calp	1:200	Mouse	Dakocytomation
Mesenchymal marker	Vimentin	V9	1:150	Mouse	Dakocytomation
(Neuro)endocrine markers	Neurofilament	2F11	1:100	Mouse	Invitrogen
	Chromogranin	NS55	1:50	Rabbit	Dakocytomation
Lymphoid markers	CD3	F7238	1:50	Mouse	Dakocytomation
	CD20	L6	1:800	Mouse	Leica Biosystems

Virtually all neoplastic cells showed a strong and diffuse membranous and cytoplasmic immunolabelling for PanCK. In addition, the same cells also showed a diffuse cytoplasmic positivity that ranged from mild to intense for CK 5/6, while mild cytoplasmic positivity for CK 8/18 was observed in scattered neoplastic cells. Occasionally, tumour cells showed a nuclear immunolabelling for p63. Interestingly, aggregates of p63-positive cells were observed specifically adjacent to, and palisading on connective stromal septa. Calponin immunoreactivity was only observed in stromal cells and on vessel walls. Vimentin expression was limited to tumour stromal cells, infiltrating inflammatory cells (lymphocytes and histiocytes) and blood vessels. Immunolabelling for neurofilaments was only observed in the peripheral nerves within tumour nodules, whilst chromogranin was negative. Lymphocytic aggregates at the periphery of the tumour had an equal distribution of CD3+ and CD20+ cells, whilst tumour infiltrating lymphocytes were mainly CD3+ (Fig. 4).

In the light of the IHC results, the tumour was deemed an undifferentiated laryngeal carcinoma.

In order to better clarify the content of the large cytoplasmic vacuoles that were consistently observed in both cytological and histological samples, a periodic acid-Schiff reaction (PAS) was performed. However, both on cytological and histological samples, cytoplasmic vacuoles were PAS-negative.

**Fig. 5:**
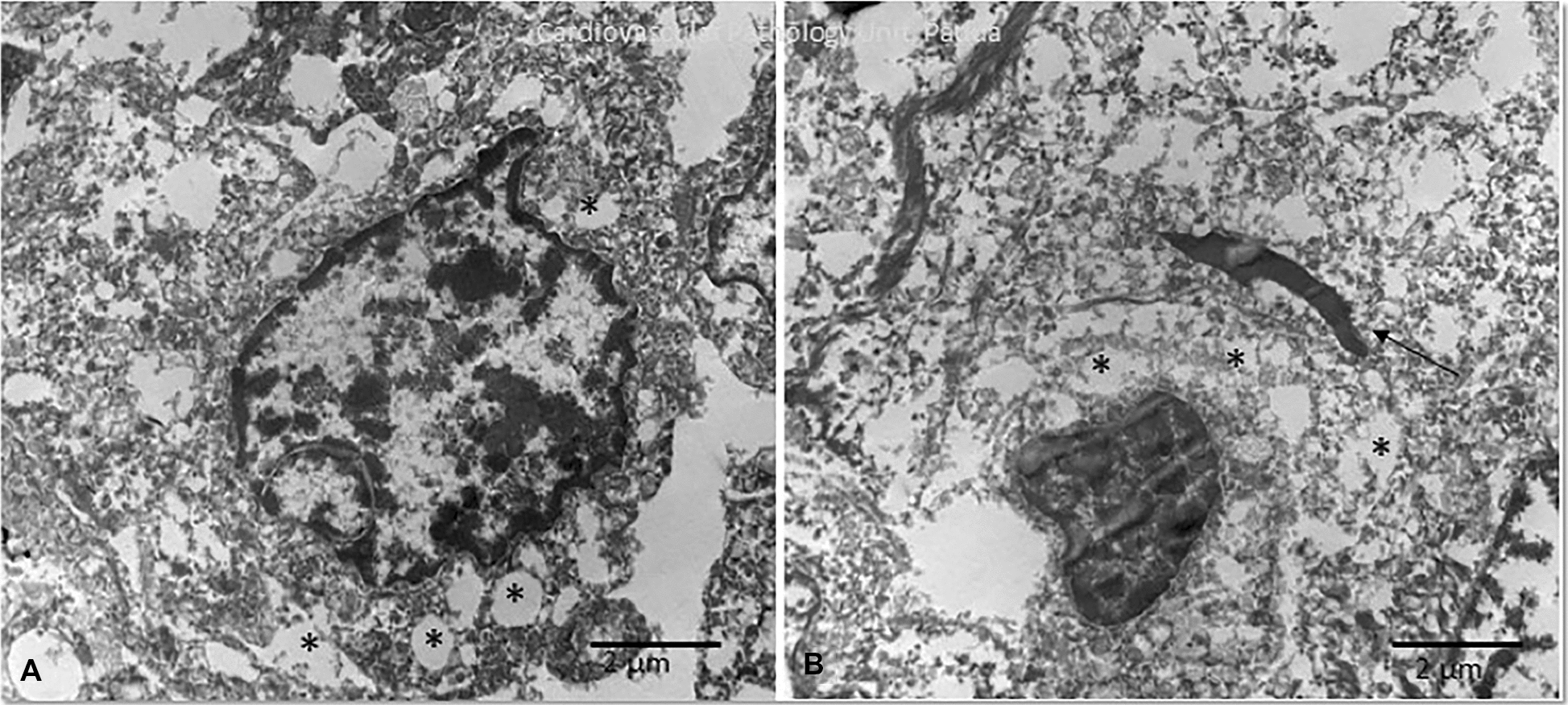
Transmission electron microscopy: **A**, **B** ultrastructural image of undifferentiated neoplastic cells with empty cytoplasmic vacuolations (asterisks). In many cells, cytoplasmic electron-dense deposits were also observed (**B**, arrow)

Neoplastic tissue was then excised from paraffin and processed for transmission electron microscopy (TEM) examination. Ultrastructural results confirmed the presence of pleomorphic neoplastic cells with cytoplasmic vacuolations. Moreover, the presence of cytoplasmic, non-membrane bound, electron-dense deposits was also observed (Fig. 5). TEM results therefore confirmed the diagnosis of undifferentiated laryngeal carcinoma, with the presence of cytoplasmic accumulations of electron-dense material that was compatible with the eosinophilic bodies observed both on cytology and histopathology.

## Discussion and conclusions

In this study, we describe an undifferentiated laryngeal carcinoma in a cat characterized by eosinophilic perinuclear bodies compatible with hyaline granules.

Laryngeal tumours are extremely rare in cats. Due to their low prevalence, studies describing feline laryngeal tumours are limited to case reports or case series in which neoplasms are often grouped with other laryngeal diseases [2–6]. In studies describing laryngeal disease in cats, the incidence of primary laryngeal neoplasms ranges from 28.5 to 34.7% [2, 3]. In a study describing masses located in the larynx and in the trachea, neoplastic diseases accounted for 77.8% [4]. According to the literature, lymphoma is the most commonly diagnosed primary neoplasm of the larynx in cats, with squamous cell carcinoma being the second most common tumour in this location [2–7]. Other tumour types such as adenocarcinoma and undifferentiated round cell tumour have only been reported in very few cases [2–4].

In our case, cytological findings were strongly suggestive of malignant neoplasia, but, due to the marked cellular pleomorphism, the scarce differentiation, and the unexpected presence of the large eosinophilic perinuclear bodies a definitive diagnosis could not be achieved. Histopathology confirmed the presence of a malignant highly pleomorphic tumour with large eosinophilic perinuclear bodies. Immunohistochemical examination confirmed the diagnosis of carcinoma due to a diffuse and strong expression of PanCK by neoplastic cells, and the lack of positivity for mesenchymal and neuroendocrine markers. Further extension of IHC panel showed diffuse, mild to intense positivity for CK5/6 (basal epithelial and myoepithelial marker), whilst CK8/18 (luminal epithelial marker) was only expressed in scattered neoplastic cells. This allowed for further inference about the nature of the tumour that was deemed an undifferentiated carcinoma with a basal phenotype, confirmed by the p63 positivity of neoplastic cells resting on collagenous septa [9].

The striking microscopic feature of this tumour, that was consistently observed in both cytological and histological samples, was the presence of variably sized, well demarcated, intensely eosinophilic, glassy, round, PAS-negative cytoplasmic bodies more often found in the perinuclear area. Similar structures have been described in human pathology in several tumour types, as well as non-neoplastic diseases, and are referred to as hyaline granules (HGs) or hyaline bodies [10–14]. Regardless of the tumour of origin, HGs seem to have a heterogeneous origin. Histological, immunohistochemical, and ultrastructural investigations conducted on different tumour types have revealed HGs as accumulations of granular or amorphous secretory material within dilated rough endoplasmic reticulum, Golgi apparatus, or non-membrane bound structures, swollen mitochondria, accumulations of intermediate filaments or of components of the basement membrane [10, 12]. In humans, HGs have been reported in 8 to 50% of cases of hepatocellular carcinoma (HCC) and their presence is considered of great diagnostic significance for primary and metastatic lesions, while their prognostic value is still debated. More specifically, a study describing these entities in cytological samples of human HCC found that HGs were associated with the granular cellular type, but failed to demonstrate an association between the presence of HGs and tumour grade [10], whilst other studies reported the association of HGs with more differentiated HCCs [11]. On the contrary, in a recent report, the presence of HGs on histology was associated with shorter overall survival in patients with HCC [12]. HGs have also been extensively described in renal cell carcinomas (RCCs) and oncocytomas in human patients [10, 13, 14]. HGs have been described in up to 17% of RCCs, can greatly vary in size (reported range 1–30 μm), can be positive or negative to PAS staining, and are more commonly composed of basement membrane material that is secreted and accumulated within rough endoplasmic reticulum or membrane-bound organelles [10, 13, 14]. More specifically, HGs are more commonly associated with granular and mixed granular-clear cell RCCs, while they are considered an exclusion criterion for the diagnosis of chromophobe type of RCC and oncocytoma of the kidney [10]. As for HCC, HGs are found in both primary and metastatic RCC lesions [10].

In our study the large eosinophilic cytoplasmic bodies were observed in both cytological and histological samples. Their microscopic features, as well as their ultrastructural appearance, were compatible with those observed for HGs in human cancer. Unfortunately, immunohistochemical analysis failed to further characterise these cytoplasmic bodies, which were negative for CK8/18. In fact, a subset of HGs in HCC can have the ultrastructure of Mallory bodies and are described as accumulations of intermediate filaments (as well as sequestrosome 1/p62 and ubiquitin) consistently positive for CK8 and CK18 [10–12]. Moreover, hyaline bodies in our case were diffusely PAS-negative on both cytological and histological samples. HGs found in RCC are usually PAS-positive but a subset of HGs in renal tumours, as well as in other neoplasms, can be PAS-negative [10, 13, 14]. In veterinary medicine, hyaline bodies have been reported in a case of HCC in a dog [15]. In their case, Masserdotti et al. [15] described these entities as round to polygonal, glassy, eosinophilic intracytoplasmic bodies in cytological and histological specimens. As in our case, a wide IHC panel failed to identify the content of these bodies which were focally PAS positive. Ultrastructural analysis identified these deposits within the rough endoplasmic reticulum or its remnants, therefore strengthening the hypothesis of these bodies being accumulations of proteinaceous material. Based on microscopic and ultrastructural appearance we can also infer that the eosinophilic bodies found in our case are composed of deposits of proteinaceous material possibly aberrantly produced by neoplastic cells.

To the best of our knowledge this is the first report of undifferentiated laryngeal carcinoma in a cat. Our case confirms the clinical features and the negative prognosis of feline laryngeal tumours. Moreover, for the first time in feline tumours, we identified structures compatible with HGs on both cytology and histology and we described their microscopic, immunohistochemical, and ultrastructural features. Future investigations are warranted to evaluate the presence of HG-like structures in feline tumours and their diagnostic and prognostic value.

## Data Availability

All data generated or analysed during this study are included in this published article and its Additional files.

## Abbreviations

BCS: Body condition score
IHC: Immunohistochemistry
PanCK: Pancytokeratin
CK: Cytokeratin
PAS: Periodic acid-Schiff reaction
TEM: Transmission electron microscopy
HGs: Hyaline granules
HCC: Hepatocellular carcinoma
RCCs: Renal cell carcinomas
